# Complete Genome Sequence of Multidrug-Resistant *Citrobacter freundii* Strain P10159, Isolated from Urine Samples from a Patient with Esophageal Carcinoma

**DOI:** 10.1128/genomeA.01754-15

**Published:** 2016-02-18

**Authors:** Xiaodong Liu, Yong Huang, Xiaomeng Xu, Yachao Zhao, Qiang Sun, Zhiyi Zhang, Xianglilan Zhang, Yi Wu, Jie Wang, Dongsheng Zhou, Xiaoping An, Guangqian Pei, Yunfei Wang, Zhiqiang Mi, Zhe Yin, Yigang Tong

**Affiliations:** a Anhui Medical University, Hefei, People’s Republic of China; b State Key Laboratory of Pathogen and Biosecurity, Beijing Institute of Microbiology and Epidemiology, Beijing, People’s Republic of China

## Abstract

*Citrobacter freundii* is an opportunistic pathogen that can cause diarrhea, septicemia, meningitis, and urinary tract infections. We report here the complete genome sequence of *C. freundii* strain P10159, isolated from urine samples from a patient in China with esophageal carcinoma. The genome has 5,080,321 bp and 4,768 coding sequences, with a G+C content of 51.7%.

## GENOME ANNOUNCEMENT

*Citrobacter freundii* a Gram-negative, aerobic, and short-rod bacterium and is commonly present in the gut microbiota in humans and other animals (1). It is an opportunistic pathogen that causes diarrhea, septicemia, meningitis, and urinary tract infections, especially in immunocompromised people (2–4). However, with the abuse of antibiotics, *C. freundii* has become resistant to common antibiotics (5), causing great challenges to the clinical treatment of infections.

We have sequenced the whole genome of a *C. freundii* strain isolated from the urine samples from a patient with esophageal carcinoma in China. Sequencing was carried out using the Ion Torrent Personal Genome Machine (Life Technologies, USA). Library preparation, sequencing reactions, and runs were performed according to the manufacturer’s instructions. The high-quality 2,177,098 shotgun sequencing reads and 852,601 mate-pair sequencing reads were used to assemble the whole genome using the GS Assembler software (Newbler) version 2.9.1, resulting in 14 scaffolds. The *N*_50_ scaffolding size was 3,769 Kbp, which is the same size as the largest scaffold. The complete genome sequence of *C. freundii* P10159 is 5,080,321 bp, with a G+C content of 51.7%.

Annotation was performed using the Bacterial Annotation System (BASys) (6) and Rapid Annotations using Subsystems Technology (RAST) (7) online servers and modified manually. The genome contained 4,768 predicted protein-coding sequences (CDSs), 24 rRNAs, and 69 tRNAs. In subsystem distribution of the annotation genome, 719 genes were involved in carbohydrate metabolism, 304 genes were involved in protein metabolism, 157 genes were involved in fatty acids, lipids, and isoprenoids, 52 genes were involved in phosphorus metabolism, 119 genes were responsible for virulence, disease, and defense, and 44 genes were associated with phages, prophages, transposable elements, and plasmids. *C. freundii* CAV1741 (accession no. CP011657), *C. freundii* CAV1321 (accession no. CP011612), and *C. freundii* CFNIH1 (accession no. CP007557) were the closest neighbors to strain *C. freundii* P10159, with identities of 96%, 96% and 90%, respectively. The orthologous genes and unique genes among the four genomes were identified and counted using the Pan-Genomes Analysis Pipeline (PGAP) under the defect parameter (8). Those four genomes shared 3,395 CDSs in total. Strain P10159 shared 3,613, 3,606, and 3,488 orthologous CDSs with CAV1321, CAV1741, and CFNIH1, respectively. In addition, 787 CDSs from the P10159 genome were classified as unique, followed by 650 CDSs from CHNIH1, 48 CDSs from CAV1741, and 16 CDSs from CAV1321.

To gain a clear understanding of the genomic basis for the observed antibiotic resistance traits, the genome was searched for specific genes known to confer antibiotic resistance. The result shows some antibiotic resistance genes in the genome conferred resistance against some of the tested antibiotics. Genes, such as *parC*, *parE*, and *gyrA*, were detected in the genome, showing resistance to fluoroquinolones; the β-lactamase gene *ampC*, the MATE family of multidrug resistance (MDR) efflux pumps, and multidrug major facilitator super family (MFS) genes were also found in the genome.

In conclusion, we present the complete genome sequence of *C. freundii* P10159. Further genome analysis of *C. freundii* strains will allow a better understanding of the resistance mechanisms and aid in therapeutic agent development in the future.

### Nucleotide sequence accession number.

The complete genome sequence has been deposited in the NCBI database under the accession no. CP012554. The version described in this paper is the first version.

## References

[1] Guerrant RL, Dickens MD, Wenzel RP, Kapikian AZ 1976 Toxigenic bacterial diarrhea: nursery outbreak involving multiple bacterial strains. J Pediatr 89:885–891. doi:10.1016/S0022-3476(76)80591-4. 792408

[2] Pardia SN, Verma IC, Deb M, Bhujwala RA 1980 An outbreak of diarrhea due to *Citrobacter freundii* in a neonatal special care nursery. Indian J Pediatr 47:81–84. doi:10.1007/BF02900180. 7216341

[3] Schmidt H, Montag M, Bockemühl J, Heesemann J, Karch H 1993 Shiga-like toxin II-related cytotoxins in *Citrobacter freundii* strains from humans and beef samples. Infect Immun 61:534–543. 8423084 10.1128/iai.61.2.534-543.1993 PMC302761

[4] Joaquin A, Khan S, Russel N, Al Fayez N 1991 Neonatal meningitis and bilateral cerebellar abscesses due to *Citrobacter freundii*. Pediatr Neurosurg 17:23–24. doi:10.1159/000120561. 1811708

[5] Nada T, Baba H, Kawamura K, Ohkura T, Torii K, Ohta M 2004 A small outbreak of third-generation cephem-resistant *Citrobacter freundii* infection on a surgical ward. Jpn J Infect Dis 57:181–182. 15329453

[6] Van Domselaar GH, Stothard P, Shrivastava S, Cruz JA, Guo A, Dong X, Lu P, Szafron D, Greiner R, Wishart DS 2005 BASys: a Web server for automated bacterial genome annotation. Nucleic Acids Res 33:W455–W459. doi:10.1093/nar/gki593. 15980511 PMC1160269

[7] Aziz RK, Bartels D, Best AA, DeJongh M, Disz T, Edwards RA, Formsma K, Gerdes S, Glass EM, Kubal M, Meyer F, Olsen GJ, Olson R, Osterman AL, Overbeek RA, McNeil LK, Paarmann D, Paczian T, Parrello B, Pusch GD 2008 The RAST server: Rapid Annotations using Subsystems Technology. BMC Genomics 9:75. doi:10.1186/1471-2164-9-75. 18261238 PMC2265698

[8] Zhao Y, Wu J, Yang J, Sun S, Xiao J, Yu J 2012 PGAP: Pan-Genomes Analysis Pipeline. Bioinformatics 28:416–418. doi:10.1093/bioinformatics/btr655. 22130594 PMC3268234

